# Complex functions of human lateral frontopolar cortex

**DOI:** 10.1093/brain/awaf289

**Published:** 2025-08-07

**Authors:** Bolton K H Chau, Chun-Kit Law, Jocelyn Y L To, David H K Shum, Rogier B Mars

**Affiliations:** Department of Rehabilitation Sciences, The Hong Kong Polytechnic University, Hung Hom, Hong Kong; University Research Facility in Behavioral and Systems Neuroscience, The Hong Kong Polytechnic University, Hung Hom, Hong Kong; Mental Health Research Centre, The Hong Kong Polytechnic University, Hung Hom, Hong Kong; Department of Rehabilitation Sciences, The Hong Kong Polytechnic University, Hung Hom, Hong Kong; Department of Rehabilitation Sciences, The Hong Kong Polytechnic University, Hung Hom, Hong Kong; Department of Rehabilitation Sciences, The Hong Kong Polytechnic University, Hung Hom, Hong Kong; Mental Health Research Centre, The Hong Kong Polytechnic University, Hung Hom, Hong Kong; Research Institute for Smart Ageing, The Hong Kong Polytechnic University, Hung Hom, Hong Kong; Wellcome Centre for Integrative Neuroimaging, Centre for fMRI of the Brain (FMRIB), Nuffield Department of Clinical Neurosciences, John Radcliffe Hospital, University of Oxford, Oxford OX3 9DU, UK; Donders Institute for Brain, Cognition and Behaviour, Radboud University Nijmegen, Nijmegen 6525 HR, The Netherlands

**Keywords:** frontopolar cortex, area 10, anterior prefrontal cortex, neuropsychology, decision making

## Abstract

Recent anatomical studies have shown that, compared to other primates, the human frontal pole (FP) contains a unique lateral subdivision (FPl). This area provides an important target for understanding the uniqueness of human intelligence. Paradoxically, patients with FP lesions often perform normally on standard neuropsychological tests, while experiencing problems in real-life or simulated situations.

This paper aims to review the complex functions of the FPl that may account for the dysfunctions observed in these patients. First, we consider studies of FP lesion patients that reveal deficits in analogical reasoning and prospective memory. Second, we review, mainly based on neuroimaging and neurostimulation studies, the FPl’s involvement in exploratory decision making, information integration and the representation of abstract rules. We argue that these functions primarily stem from the FPl’s capacity to manage multiple sources of information and to reduce that information into simpler features for guiding behaviour. Finally, we propose a model of the FPl that emphasizes its role in decomposing high-dimensional information to enhance decision making processes in conjunction with connected regions, including the posterior cingulate cortex, anterior cingulate cortex, and dorsolateral prefrontal cortex.

## Teaser

The lateral frontal pole of the human brain is unique among primates. However, patients with damage to this area often perform normally on neuropsychological tests. Chau *et al.* review findings from anatomy, neurology, and cognitive neuroscience, and propose that the lateral frontal pole enhances decision making by decomposing information.

## Introduction

The frontal pole (FP) of the human cortex has greatly expanded compared to that of other primates and contains a lateral subdivision that is unique to the human brain.^1,2^ Paradoxically, neurological patients with a lesion in the FP often perform normally on standard neuropsychological tests.^3-5^ In contrast, functional neuroimaging studies are able to detect task-related activations of the FP, showing that the lateral part of FP often co-activates with a network of specific other brain regions.^6,7^ These observations suggest that either the FP serves functions that can easily be compensated for, or—most likely—that standard neuropsychological tests are insensitive to specific deficits accompanying FP damages. In this review, we briefly explain the unique anatomical features of the human FP. We emphasize the human lateral subdivision of FP (FPl) whenever possible because it is particularly expanded in the human brain. However, in other situations, we discuss the overall FP when it is not possible to make specific claims about the FPl, such as in patient studies involving extended lesions. We then highlight the observations in patients with lesions including the FPl and contrast them with the wealth of functional activations seen in this area. Finally, we argue that functions ascribed to the FPl generally involve managing multiple sources of information, decomposing high-dimensional data into simpler features, or both, and propose a model suggesting how the FPl interacts with other closely connected regions to augment decision making.

## Anatomical features of the human FPl

**Figure 1:**
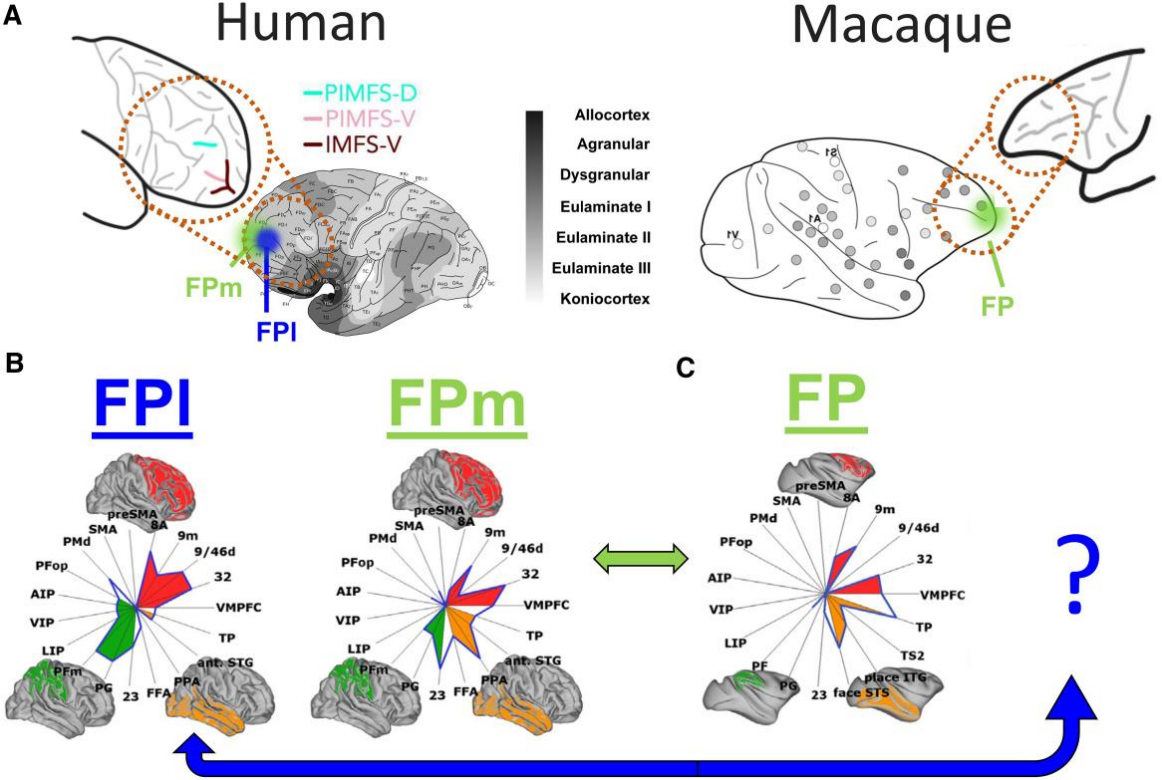
**Anatomical features of the FP.** (**A**) The human FP is greatly expanded compared to the macaque FP. Comparing the sulcal anatomy across species showed additional sulci in the lateral surface of the human FP, which are absent in the macaque (*insets*).^11^ These additional sulci are dorsal and ventral paraintermediate frontal sulcus (PIMFS-D and PIMFS-V, respectively) and vertical rami of the intermediate frontal sulcus (IMFS-V). (**B**) Parcellation based on cell staining or connectivity profiles further separates the FP into lateral and medial subdivisions (i.e. FPl and FPm, respectively). (**C**) The macaque contains a similar FPm; however, the FPl appears to be unique in the human. Adapted from Amiez *et al*.,^11^ Neubert *et al*.^12^ and Garcia-Cabezas *et al*.^13^ FPl = frontal pole.

The human FP is often equated with Brodmann’s area (BA) 10 at the most rostral end of the prefrontal cortex. Human BA10 is disproportionately large, occupying 14% of brain volume compared with 2%–3% in other great apes.^8^ Although it has been argued that allometrically speaking, this increase is modest,^9^ it means that the absolute number of neurons in this part of the brain is substantially greater than in any other species. Early comparative neuroimaging studies between macaques and humans investigated the connectivity of the FP, reporting increased functional connectivity between its lateral part and parts of the inferior parietal cortex that are also known to be expanded in the human brain.^10^ Together, these results suggested that the human FP is more expanded and might have an additional subdivision in the human brain, compared to that of other primates (Fig. 1).

Early cytoarchitectonic maps of the human FP generally defined it laterally as the most rostral end of the rostral part of the superior frontal gyrus and a small part of the middle frontal gyrus, anterior to area 46. On the medial surface, it was taken to the border area BA32 rostral to the cingulate gyrus.^14,15^ A more recent map extended more caudally on the medial surface and suggested a number of subdivisions.^16^ This led Bludau and colleagues^17^ to perform an observer-independent parcellation based on cell staining. They identified a lateral (FP1) and medial (FP2) subdivision, with distinct functional and connectivity profiles. Simultaneous connectivity-based parcellations based on neuroimaging data reported similar medial FPm and lateral FPl divisions (Fig. 1B).^12,18^ Importantly, the connectivity profile of the FPl seemed to be unique to the human brain, with no preferential match identified in the macaque brain (Fig. 1C).^12^ More recent work comparing sulcal anatomy in a larger sample of primates also concluded that the FPl is different between humans and monkeys, as the human FPl contains additional sulci that are absent in monkeys (Fig. 1A, insets), although the functional implications of having these additions are unclear.^11^ Similar sulcal patterns to those in humans are found in chimpanzees and great apes as well.

## FP lesion patients often perform normally on neuropsychological tests but experience real-life problems

Given that the FPl contains so much potentially novel territory in the human brain, one would expect it to have a distinct role in higher-order cognition. However, detecting the precise deficits in patients with a FPl lesion is not straightforward, since many of them appear to perform normally on standard neuropsychological tests. For example, Hoffmann and Bar-On^3^ reported a rare case of a patient with a focal FP lesion. This patient performed normally on many neuropsychological tests, some of which are supposed to be sensitive to frontal lobe lesions, such as the Wisconsin Card Sorting Test (WCST), the Tower of London, the Trail Making Test, the Rey-Osterreith Complex Figure Test, the Frontal Systems Behavioral Scale and the Emotional Quotient Inventory. Since there are very few reported cases of focal FPl lesions, here we broaden our discussion slightly to consider cases with lesions that extend to the FPm or other neighbouring frontal regions.

One example is a patient who had anterior frontal damage including the FP. Similar to the patient described by Hoffmann and Bar-On,^3^ Goel and Grafman^19^ reported a patient who showed average or above-average performance on a wide range of neuropsychological tests. He used to be an architect, but after the damage, he was unable to generate designs, and as a result, he was forced to retire. When he was tested on a task simulating real-life interior design, although he was able to use his prior knowledge to structure the problem, he failed to make further progress in generating solutions. Another study that included 18 patients with a FP lesion showed similar results.^20^ These patients generally showed normal performance when tested on a standard and well-structured planning task (namely the Delis-Kaplan Executive Function System Tower Task). However, they showed poor planning when tested on a real-life planning task that required them to make travel plans within certain limits (e.g. cost and time) for an imaginary couple.

Similarly, an early study by Shallice and Burgess^21^ tested three patients with an extended lesion in the frontal lobe. All of them showed above-average levels of intelligence, scoring above 112 in both the verbal and performance IQ subscales of the Wechsler Adult Intelligence Scale. However, they performed particularly poorly on two tasks that lack clear structure, including the Six Element Test, which requires participants to complete six small tasks in their own preferred order, and the Multiple Errands Test, which requires them to complete tasks by navigating in a real shopping area.

These findings illustrate that standardized tests are sometimes insensitive to capturing functions or dysfunctions of the FP. What is common to the above patients is that they perform poorly on tasks that are naturalistic, information-rich and/or poorly structured, while their IQ, as well as some other neuropsychological test scores, are in the normal range.

## FP lesion patients show deficits in analogical reasoning and prospective memory

FP lesion patients often perform poorly on complex tasks simulating real-life problems or have difficulties with actual real-life situations. These problems are often characterized by their complex and disorganized structures. This may explain why they also perform poorly in complex cognitive tasks, such as those assessing individuals’ analogical reasoning or prospective memory, which we review next.

One suggestion for the cause of FP lesion patients’ real-life problems is related to their deficits in analogical reasoning, the ability to identify similarities between distinct situations or stimuli and to generalize them to new cases.^22-24^ Urbanski and colleagues^23^ developed a matching task to assess analogical reasoning, in which patients are given an exemplar set of three letters. The letters may contain different features, such as different colours, fonts and sizes. Patients are given two alternative sets of three letters and asked which one is a better match. For example, the exemplar may look symmetrical by having the first and third letters share the same colour and size, and patients should choose an alternative that looks symmetrical. The crucial element of the task is that patients must identify abstract features that the target shares with the exemplar. It was found that those with a lesion that included the FPl, compared to those with a lesion outside the FPl, performed poorly in this analogical reasoning task. These findings are consistent with neuroimaging studies showing FPl activity is related to analogical reasoning in both social and non-social contexts.^22,25,26^

Another suggestion for the cause of FP patients’ real-life problems is that they often show impairment in prospective memory, the ability to remember to do things in the future. Volle and colleagues^27^ tested 45 patients with a variety of brain lesions on a task that required prospective memory to perform an action either at a specific time or for a specific event. They found that those with an FP lesion were impaired when prospective memory was required for time-specific responses. A closer inspection suggests that the specific FP lesion associated with the impairment was located at the border between the FPl and FPm, according to the connectivity-based parcellation atlas.^12^ This is consistent with brain imaging data showing signals in a similar FP region associated with prospective memory, where the technique allows for more precise localization of the FPl’s involvement in maintaining future intentions.^28^ Patient Z.P., who had a lesion in a similar area, was assessed for his prospective memory.^29^ When assessed by neuropsychological tests, he showed a poor prospective memory score, while the scores in retrospective memory and general intelligence were average or above average. Patient Z.P. was tested on a task requiring two sets of actions to be memorized and one of the two sets reproduced after performing another task in between. Interestingly, although he was able to recall both sets of actions, he failed to recall which set of actions he was asked to reproduce. Furthermore, in a different situation, Patient Z.P. was instructed to respond to a target cue that was occasionally presented while concurrently performing a different task. Although he failed to make these responses more often than the controls, he was able to recall the instructions of this task accurately. Taken together, these findings suggest that Patient Z.P., as well as other FP lesion patients, appeared to exhibit deficits in specific aspects of prospective memory. They were capable of retaining the specific content of prospective memory; however, they had deficits in enacting the content according to the specific time or event.

In summary, these lesion studies suggest that the FPl has specific functions in solving problems with complex structures, such as analogical reasoning and prospective memory. Next, we review neuroimaging studies that show activity in the FPl and contrast its role with other brain regions in specific tasks.

## Functional activity of the FPl during decision making tasks

Neurological studies thus far show that patients with FP damage often exhibit normal behaviour on standard neuropsychological assessments but have severe deficits in real-life tasks that are characterized by poor structure. This might be due to difficulties in higher-level abilities, such as analogical reasoning and prospective memory. Interestingly, these higher-level abilities could be linked to complex decision making tasks, which increasingly aim to capture naturalistic behaviours^30^ and tend frequently to show activation of the FP. In these tasks, the FP seems to interact with specific networks of other brain regions. Here, we review some of these paradigms.

Neuroimaging data suggest that the FPl co-activates with other prefrontal and cingulate regions in a wide range of cognitive functions.^31,32^ The FPl is often considered part of the fronto-parietal network, based on parcellation of resting-state functional MRI (fMRI) data,^33,34^ and is often compared with more posterior areas in the lateral frontal cortex.^1,35^ In addition, the FPl is functionally connected with the anterior cingulate cortex (ACC) and posterior cingulate cortex (PCC), based on seed-based functional connectivity analysis,^12,36,37^ and is frequently co-activated with the PCC or ACC depending on the specific task.^38^ It is worth mentioning that several recent macaque studies have also shown connectivity patterns between the FP and cingulate cortex,^39-41^ although our focus is on the human-unique FPl. Next, we review the roles of the FPl during decision making and discuss its interactions with the ACC and PCC.

## Exploratory decisions and counterfactual information

A number of studies have implicated the FPl in exploratory decision making. In a classic study, Daw and colleagues^42^ asked participants to perform a four-arm bandit task while undergoing fMRI. When participants forwent the most rewarding option and explored other alternatives, the FPl became particularly active. Subsequent studies showed that the FPl not only shows a step change in activity during exploration, but it also parametrically tracks counterfactual information by encoding the value of unchosen alternatives.^43,44^ This counterfactual information is particularly useful for monitoring changes in the environment to guide future explorations. Interestingly, exploratory decisions could be motivated by two possible reasons—simply choice randomness (i.e. random exploration) or more strategically exploring new information to maximize future rewards (i.e. directed exploration). Inhibiting the FPl using transcranial magnetic stimulation (TMS) disrupts directed exploration but not random exploration.^45^ These findings suggest that the FPl has a causal role in directed exploration.

During directed exploration, it is important to track the uncertainty of each option and then sample the options with large uncertainties to gain new information. In Tomov and colleagues’ study,^46^ participants were required to track the reward history of two options and estimate the variability of the rewards. Interestingly, the FPl activity was related to the difference in uncertainty between the two options but not the overall uncertainty.

It has been shown that exploratory decision making is supported by more distributed neural networks beyond the FPl. Recently, a Partially Observable Markov Decision Process (POMDP) model was applied to dissociate neural signals that guide exploration and exploit immediate rewards in the human brain.^47^ The results showed that the FPl, together with other regions, was specifically involved in exploration. Furthermore, the same study compared exploratory behaviours in humans and macaques when given similar tasks and found that the two species employed a similar computational mechanism. These findings are intriguing because they lead to the question of why the human brain additionally involves the FPl during exploration.

Although few studies directly contrast exploratory signals in human and macaque brains, studies that look at these two species individually suggest that macaques rely on phylogenetically older brain regions, such as the ACC and the neighbouring dorsomedial frontal cortex (DMFC). For example, when macaques perform a learning task, the ACC stores the value of an alternative option to guide exploration.^48^ Interestingly, when human participants were asked to perform a similar task, in addition to a signal in the DMFC (which is located adjacent to the ACC), a similar signal was found additionally in the FPl, especially when the outcomes of the chosen option and unchosen alternatives were revealed simultaneously.^44^ Next, we describe the FPl’s capacity to manage multiple sources of information, enabling humans to update multiple options simultaneously to guide effective future explorations.

## Integrating multiple sources of choice information

The FPl has an important role in managing multiple sources of information to guide decision making. For example, to plan a trip, one must consider the appeal of the tourist attractions, while also assessing the travel time and costs. Goel and colleagues^49^ showed that patients with FP lesions performed poorly when given a task simulating such a real-life planning problem. Such planning requires the integration of multiple pieces of information, such as budget and time constraints. Functional neuroimaging data have shown that FPl activity ramps up when it is necessary to exercise this logical reasoning to integrate multiple pieces of information.

**Figure 2:**
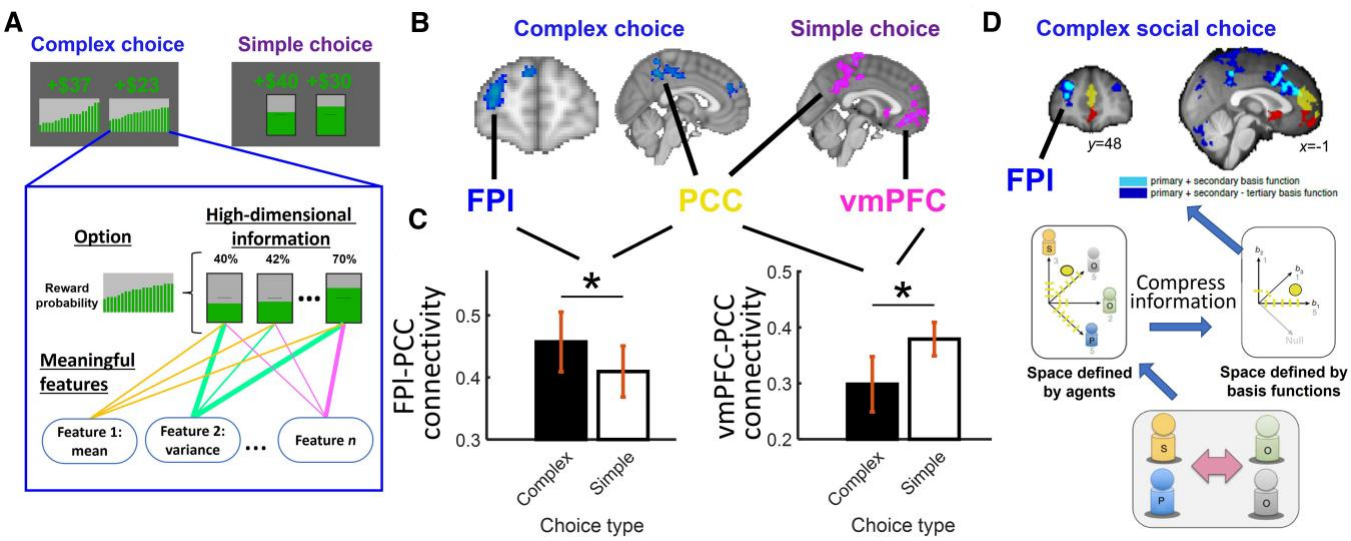
**FPl-PCC is involved in decomposing high-dimensional information**. (**A**) Complex choice often consists of high-dimensional information, which needs to be decomposed into meaning features to guide decision making. (**B**) Complex choice involved signals in the FPl and PCC, whereas simple choice involved signals in the vmPFC and PCC. (**C**) Similarly, there was stronger functional connectivity between FPl and PCC during complex choice than simple choice. In contrast, vmPFC-PCC connectivity was stronger in simple choice than complex choice. (**D**) A similar activation pattern was found in a social decision making experiment that involved processing complex information from four agents. Instead of maintaining information using a 4D space defined by the four agents, it is possible to compress the information into a 3D space defined by three basis functions. Signals in the FPl (and also other cingulate and frontal regions labelled in blue) were related to the two basis functions relevant to the decision. Adapted from Law *et al*.^50^ and Wittmann *et al*.^58^ FPl = frontal pole lateral subdivision; PCC = posterior cingulate cortex; vmPFC = ventromedial prefrontal cortex.

Sometimes it is necessary to decompose information into simpler features to guide decision making. For example, a tourist decides between destinations by considering both the average appeal and diversity of the attractions. Recently, Law and colleagues^50^ investigated neural mechanisms associated with integrating high-dimensional choice information during decision making (Fig. 2A). Participants had to choose between two complex options, each comprised of a combination of 20 gambles at different levels of probability of winning a reward, while undergoing fMRI. They showed that the FPl, as well as PCC, in the dorsal end of areas d23 and d31,^51^ carried a signal reflecting the value of these complex options (Fig. 2B and C). In contrast, when participants chose between simpler options, each of which contained a single gamble only, the FPl disengaged and the options’ values were instead reflected by the activity of the ventromedial prefrontal cortex (vmPFC) and PCC, a classic activation pattern observed when people are given relatively trivial decision making tasks.^52-54^ Although these simpler options require some degree of information decomposition, such as combining reward probabilities and magnitudes in gambling choices, vmPFC seems to be involved when such decomposition can be completed rather automatically.^55-57^ These findings showed a double dissociation in the activities of the FPl and vmPFC based on whether it was necessary to decompose complex, high-dimensional information. Future studies may elucidate, depending on the complexity of information, whether the prefrontal cortex operates via a binary switch between the FPl and vmPFC or a gradual shift between the two regions.

To investigate the roles of the FPl in complex choice, Law and colleagues^50^ applied a multivariate analysis and an artificial neural network (ANN) in combination to analyse the neural data. This approach leveraged the multi-stage hierarchical structure of the ANN to test the contributions of each brain region to each stage of decision making. In particular, the researchers first trained the ANN using human decision making data, such that the ANN made similar choices to the human participants, and then correlated different stages of the ANN with activity of various brain regions using representational similarity analysis. The study showed that, while activity of the visual cortex was related to the ANN’s lower-level input, the FPl was involved in the ANN’s intermediate processes for decomposing high-dimensional input into multiple low-dimensional features (such as the mean and variance), and the PCC was involved in integrating these low-dimensional features to guide decision making. In other words, the FPl’s role seemed to involve a dimensionality reduction of the data, which could then be processed by other brain regions. It is not clear whether the temporal structure of the task’s design contributed to the activation of the FPl in this study. FPl activity was observed in other studies that involved prospective memory or required information to be integrated across time.^27,28,59-62^ It is possible to vary the ANN further and test whether introducing a temporal structure can improve the representational similarity between the ANN and FPl.

A follow-up analysis by Law and colleagues^50^ suggested that multi-feature decomposition is an important property of the FPl. The original ANN exhibiting representational similarity to the FPl employed four filters for decomposing choice information into features that inform decision making. Interestingly, as the authors gradually reduced the number of filters to one, the ANN’s representational similarity to the FPl diminished accordingly. In other words, FPl activity appears to be linked specifically to a multi-feature, rather than single-feature, decomposition mechanism. It is unclear whether such a multi-feature decomposition process in the FPl follows the cognitive branching hypothesis by putting one process (here, decomposition of one feature) on hold and engaging in another process (here, decomposition of another feature),^63-65^ or whether such multi-feature decomposition processes occur simultaneously in the FPl. Testing these hypotheses requires techniques with high temporal resolution, such as EEG or magnetoencephalography, to scrutinize the FPl signal.

Recently, Wittmann and colleagues^58^ investigated how people decompose complex social information (Fig. 2D). Their findings suggested that individuals can decompose social information into lower-dimensional representations, which they then flexibly use to solve various social decision making problems. Interestingly, when the task required two distinct sets of lower-dimensional information to be integrated, the researchers observed an activity pattern, including the FPl, similar to that reported by Law and colleagues.^50^ Moreover, a control non-social experiment revealed a comparable signal in the FPl that involved a similar process of decomposing information.^58^

## The position of the FPl in the lateral frontal cortex’s functional hierarchy

The FPl has been examined in a wider context by considering it as part of the lateral frontal cortex and studying whether there is a functional hierarchy along the caudal-rostral axis. This generally spans across regions such as the premotor cortex, mid-dorsolateral prefrontal cortex (mid-DLPFC) and FPl.^66^ We briefly explain the Rule Abstraction model, Information Cascade model and other related literature that support this view. Evidence supporting these models generally suggests that the FPl takes an apical position in the functional hierarchy; however, we note inconsistent findings based on connectivity analyses.

**Figure 3:**
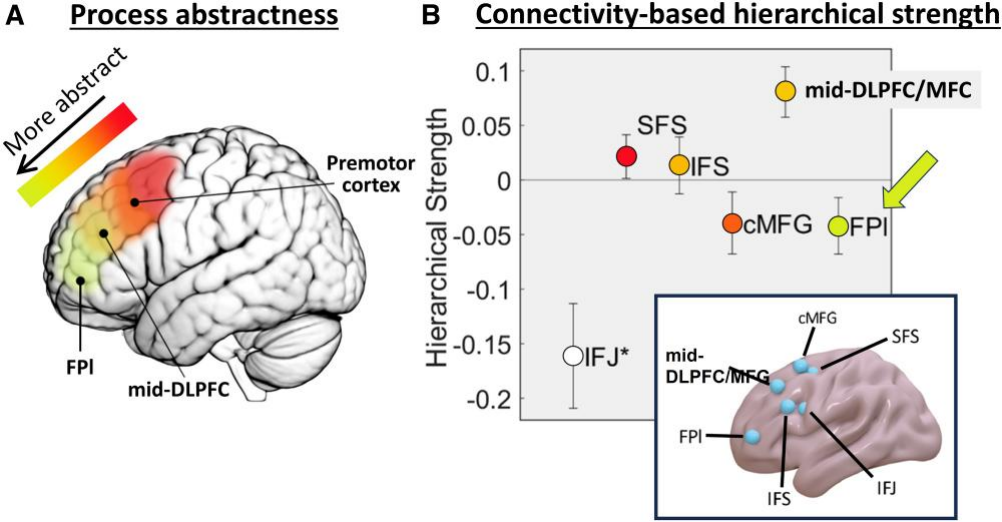
**The FPl in relation to other regions of the lateral frontal cortex.** (**A**) One approach to understanding lateral frontal cortex organization is the abstractness of the processes involved, suggesting a smooth caudal-to-rostral gradient. This suggests that, in the rostral end, the FPl is at the apex of the hierarchy for most abstract control processes. In the caudal end, the premotor cortex is involved in more concrete control processes. (**B**) A recent analysis took a different perspective on understanding lateral frontal cortex organization based on connectivity data. It assumes that a more apical region should show more efferent than afferent connections, referred to as greater hierarchical strength. As opposed to the abstractness perspective, this connectivity-based analysis showed that the region with the greatest hierarchical strength was the mid-DLPFC, instead of the FPl. These two perspectives demonstrate that the lateral frontal cortex contains multiple forms of hierarchical organization. Adapted from Pitts and Nee^74^ and Vaidya and Badre.^75^ DLPFC = dorsolateral prefrontal cortex; FPl = frontal pole lateral subdivision.

The Rule Abstraction model proposes that the lateral frontal cortex involves a caudal-to-rostral gradient of abstraction in action representations (Fig. 3A).^66^ At the caudal end, the premotor cortex is involved in controlling concrete motor plans (e.g. direct stimulus-response mapping).^67,68^ More rostrally, the mid-DLPFC is involved in controlling responses based on more abstract rules (e.g. comparing stimuli based on specific features of the stimuli).^69,70^ Furthermore, at the rostral end, the FPl is involved in controlling behaviour based on highly abstract information (e.g. frequent remapping of task rules that govern feature-to-response contingencies). This gradient also applies to tasks that require temporal control, the process of integrating past information to inform future actions. In a cognitive control paradigm involving sequential presentation of a set of stimuli, participants had to keep track of whether the stimulus series aligned with a pre-learned sequence.^71,72^ Along the lateral frontal cortex, the most caudal areas (e.g. inferior frontal junction) were found to be involved in feature control, which is to control actions based on concrete stimulus features. The middle areas (e.g. the mid-DLPFC) were involved in contextual control, which is to select actions appropriate to the abstract rule. The FPl at the rostral end of the lateral frontal cortex is involved in temporal control. This view of the FPl’s role in temporal control is in line with other findings in sequential behaviours that require tracking the temporal order of events. For example, in another task, participants were provided a series of instructions to respond to a stream of stimuli.^59,60^ FPl (as well as the premotor cortex) activity ramped up gradually over the course of executing those instructions. Nonetheless, a brain stimulation experiment showed that disrupting the FPl, but not the premotor cortex, resulted in more errors in this task. Further investigation suggested that the FPl activity was scaled by a combination of the proximity to the end of the sequence and the size of reward received.^73^ Together, these findings support the view that the FPl is involved in the abstract process of representing temporal information, whereas the posterior regions of the lateral frontal cortex are involved in more concrete action controls.

Another theory that supports the view of a caudal-to-rostral hierarchy in the lateral frontal cortex is the Information Cascade model, which emphasizes that the execution of more abstract processes relies on the bottom-up information received from the less abstract processes.^76,77^ The caudal end of the gradient encodes straightforward information for stimulus-response mapping. In contrast, the rostral end enables more abstract processes such as dynamically switching between different mapping schemes. This notion was supported by data from patients with lesions at the premotor cortex, which is implicated in concrete motor planning, who exhibited behavioural impairment across task demands from concrete to abstract,^78^ while patients with lesions at the more rostral mid-DLPFC exhibited behavioural impairment only when the task demand was abstract. Another lesion study showed consistent findings that the most abstract process was impaired regardless of whether the caudal (i.e. premotor cortex, inferior frontal gyrus) or rostral (i.e. mid-DLPFC) regions were lesioned, but the most concrete process remained intact unless the caudal region (i.e. premotor cortex) was lesioned.^79^

This caudal-to-rostral functional gradient in the lateral prefrontal cortex fits well not only with action or rule representations but also with the broader cognitive neuroscience literature. In the memory literature, the classical N-back task typically requires remembering numbers that are presented sequentially and determining whether the current number is identical to one of these previous numbers. Such paradigms are reliably associated with activity in the DLPFC (BA 9/46).^80^ However, when people are asked to memorize information with a greater degree of abstraction, such as future actions that have yet to be carried out (also known as prospective memory), the more rostral FP region is involved. This has been captured by traditional fMRI methods^28,62,81^ as well as recent mobile optical imaging methods.^61^

Similarly, during decision making, a greater degree of abstractness in choice information appears to involve more rostral regions. For example, when people are asked to choose between a large number of options, one way of solving this problem is by applying a filter to retain the more favourable options and ignore the less favourable ones. Woo and colleagues^82^ applied transcranial direct current stimulation and demonstrated that such a mechanism of filtering actual options was related to the dorsolateral prefrontal cortex. However, in other contexts, choices may require weighing different high-dimensional features and decomposing them into simpler forms. As discussed above, this involves the FPl to digest choice information when it is in large quantities.^50^ Overall, there seems to be substantial support for the idea that the lateral frontal cortex follows a caudal-to-rostral functional gradient, with the FPl located at the rostral end serving as the apex of the functional hierarchy.

Intriguingly, this view of the FPl being the apex has been challenged by studies investigating the connectivity profile of lateral frontal regions. It is argued that to affirm a functional hierarchy, regions in the higher positions should impose stronger efferent connectivity than regions in the lower positions of the hierarchy. In other words, the apical region should show the greatest magnitude in efferent connectivity and the smallest magnitude in afferent connectivity. In a series of studies, Nee and colleagues^71,72^ performed such an analysis by applying dynamic causal modelling to fMRI data.^74^ Surprisingly, the results suggested that, instead of the FPl, the mid-DLPFC is more likely to be at the apical position by showing the greatest difference in efferent versus afferent connectivity strength (Fig. 3B).^71^ Hence, the claim of a caudal-to-rostral gradient does not seem to be supported by connectivity data. Indeed, abstract processes associated with the FPl’s activity, such as temporal control, sometimes involved co-activation in the mid-DLPFC.^71,74^

Badre and Nee^35^ reconciled this controversy by suggesting that the mid-DLPFC subserves a domain-general control role, which exerts influence over the posterior regions that are involved in processes related to specific sensory-motor domains. This view is consistent with the findings that the mid-DLPFC exhibits a diverse connection profile, as shown by its connections with both sets of internally-orienting (e.g. default mode network) and externally orienting neural networks (e.g. dorsal/ventral attention networks),^83,84^ and also it plays a general role in flexibly allocating cognitive resources.^85-87^ Large scale cortical models suggest that multiple hierarchies can co-exist depending on the perspective. For example, the hierarchical organization among cortical regions varies according to the modality of sensory input,^88,89^ at least when contrasting whether the macaque brain is processing visual or somatosensory information. One possible reason for this is that, unlike the hierarchical and layered networks often seen in conventional neural network models, primate cortical regions are connected by long-range and short-range loops.^90-93^ As such, it may look as if the hierarchy of the lateral frontal regions dynamically adapts according to the specific contexts due to specific paths taken during the process.

Here, we ask: What is the function of the interaction between the FPl and mid-DLPFC during decision making? Our analysis shows that the FPl involves multiple parallel processes for decomposing high-dimensional information.^50^ In other words, the FPl contains filters to weigh each piece of information and combine them together. However, it is unclear how the filter weights are shaped according to task demands. Considering that the mid-DLPFC is involved in domain-general control processes and sometimes appears to exert top-down influence on the FPl,^35,71,72,74^ we propose that the mid-DLPFC shapes the filter weights in the FPl to facilitate the decomposition of information. This notion regarding the interaction between the mid-DLPFC and FPl can be tested by experiments that involve variable task demands, such that the same set of information needs to be decomposed in different ways.

## The FPl for augmenting decision making via multi-feature decomposition

We argue that cognitive functions associated with the FPl generally fall into two categories. The first involves managing multiple concurrent information streams. They could be from different tasks performed simultaneously, switched intermittently, or from different components of a single task. For instance, prospective memory involves remembering a future action while currently engaging in another task. Cognitive branching entails suspending one task to undertake another. Exploratory decision making considers alternative actions, while counterfactual learning requires updating information related to chosen and unchosen options. In all these cases, multiple sets of information are managed concurrently or prioritized over time. Although managing multiple information streams reliably activates the FPl, similar functions are also performed by phylogenetically older regions such as the ACC,^48,94-96^ suggesting that the FPl likely supports additional functions unique to human cognition.

The second category involves decomposing high-dimensional information into meaningful features for guiding behaviour. For example, analogical reasoning requires extracting basic components (such as colour, shape and size) from complex visuals and identifying higher-order patterns like symmetry. Directed exploration relies on summarizing past choices into measures of average reward and uncertainty, which aids in balancing exploration and exploitation. In complex decision making, individuals reduce numerous attributes into simpler features like average and variance of rewards or compressed basis functions to facilitate effective choices. These examples illustrate that the FPl is involved in decomposing high-dimensional information into meaningful features.

**Figure 4:**
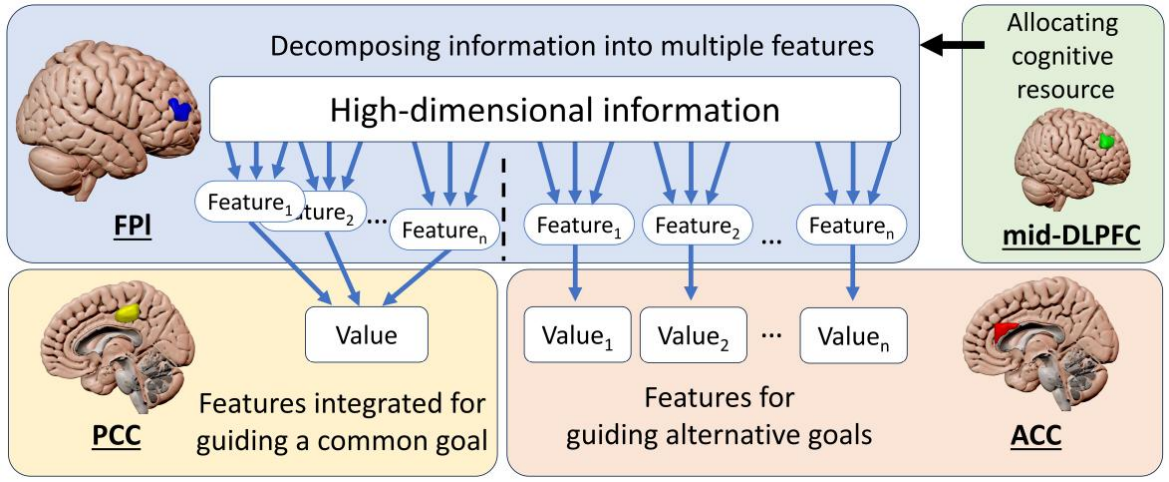
**A model of the FPl augmenting decision making via interactions with the DLPFC, PCC and ACC.** In complex environments, the FPl decomposes high-dimensional information into multiple branches of meaningful features to guide decision making. The allocation of cognitive resources for information decomposition in the FPl is modulated by the mid-DLPFC. The feature branches can either be combined via functional connectivity with the PCC to guide a common goal or, in other situations, each feature branch could be used to guide an individual goal. FPl-ACC connectivity is involved in choices between alternative goals. ACC = anterior cingulate cortex; DLPFC = dorsolateral prefrontal cortex; FPl = frontal pole lateral subdivision; PCC = posterior cingulate cortex.

We propose that the FPl plays a unique role in decomposing high-dimensional information into meaningful features to enhance adaptivity when navigating abstract and disorganized real-life problems (Fig. 4). In addition, we propose a model suggesting that the FPl achieves these functions by interacting with regions including the PCC, ACC and DLPFC.^50,65,97^ Slightly posterior to the FPl, the mid-DLPFC is involved in defining action goals, possibly shaping the filters for decomposing the features in the FPl via a top-down modulation.^85^ The FPl achieves its contributions, among others, through interactions with two cingulate regions, the PCC and ACC, two phylogenetically older regions that are involved in decision making processes in human and non-human primates. The FPl-PCC connection is particularly important when it is necessary to combine multiple features to serve a common goal. For example, for a given set of high-dimensional information, the mean and variance are decomposed independently, which are further combined to guide decision making.^50^ In contrast, the FPl-ACC connectivity is involved in scenarios when multiple goals are monitored simultaneously. For instance, exploratory behaviours involve deciding between alternative courses of actions; the value of each action is estimated separately, such that multiple action values can be updated concurrently. In this model, we propose that the FPl decomposes high-dimensional choice information into features and interacts with either the ACC or PCC, depending on whether the features inform different decisions or the same decision.

This model could be tested using paradigms that require making decisions based on high-dimensional information. Sometimes the information needs to be decomposed to differentiate which is the more valuable option, a process that may involve FPl-ACC connectivity. Sometimes the information needs to be combined to generate an overall value of a single option, a process that is supposed to involve FPl-PCC connectivity. By combining neuroimaging and patient testing, it is possible that these kinds of paradigms could eventually be developed into neuropsychological tests to assess FPl-cingulate disconnections in neurological patients.

## Final remarks

We conclude by highlighting two key questions. First, what are the specific deficits in patients with FPl lesions? These patients experience real-life problems, such as difficulties in their career, financial planning and marriage,^19,21,29,98,99^ but perform normally in many conventional neuropsychological tests or cognitive tasks that are more structured and concrete. For example, while patients reported by Goel and colleagues^20^ were impaired in a simulation of real-life travel planning, they showed normal performances in standard tower tests that assess planning. Intriguingly, reminiscent of studies relating the FPl to analogical reasoning,^22,23,25,26^ recently Mole and colleagues^100^ tested a large cohort of 247 patients with diverse lesion sites. They found that impairments on an analogical reasoning task were instead related to damage to a frontal network posterior to the FPl. Although the reasons underlying the discrepant findings are unclear, it has been argued that Mole and colleagues’ task primarily required identifying analogy rather than exercising reasoning—applying the knowledge of analogy to solve new problems.^101^ This aligns with our proposal that the FPl has a function in decomposing high-dimensional information into features for guiding deliberation of choices.

Second, what cognitive capabilities have emerged with the evolution of the human FPl? Addressing this question requires considering the unique anatomical features of the FPl, as described earlier in this paper. For example, the human FPl connects to the rest of the brain by a unique pattern not found in other primates, such as macaques. However, how this FPl-centred functional network supports cognitive abilities remains unexplored. Considerable neuroimaging studies have investigated the relationships between functional networks, such as the default mode network, and cognitive functions.^102^ These have provided the necessary analytic techniques for investigating the contributions of these uniquely human, FPl-centred functional networks to cognitive capabilities.

Finally, while it is evident from everyday experience that humans behave differently from other primate species, it remains surprisingly difficult to identify specific cognitive abilities that are uniquely human. Previous candidates, such as communication, tool use and abstraction, have been challenged or found to exist in other species to some extent.^103^ Uncovering the distinct contributions of the FPl to human cognition is a key challenge, but doing so may provide crucial insights into the cognitive capacities that set humans apart.
